# Surveillance of zoonotic pathogens in cattle and dromedaries sacrificed at the Grand Magal of Touba: a cross-sectional survey

**DOI:** 10.1016/j.nmni.2026.101714

**Published:** 2026-01-27

**Authors:** Ihssane Ouaddane, Coumba Diouf, Georges Diatta, Adama Zan Diarra, Mbayang Faye, Cheikh Sokhna, Philippe Gautret

**Affiliations:** a Aix Marseille Univ, AP-HM, SSA, RITMES, IRD, MINES, Marseille, France; b IHU-Méditerranée Infection, Marseille, France; c Aix Marseille Univ, IRD, AP-HM, SSA, Campus International IRD-UCAD de L’IRD, Dakar, Senegal; d EMR 279, IRD, MINES, Campus International IRD-UCAD de Hann, Dakar, Senegal

**Keywords:** Zoonotic surveillance, Mass gatherings, Tick-borne diseases, Livestock, Grand Magal of Touba

## Abstract

**Introduction:**

Mass gatherings increase infectious disease risks through human, environmental, and zoonotic pathways. The Grand Magal of Touba (GMT) involves the annual sacrifice of animals under limited biosafety, yet data on zoonotic pathogens are scarce. This cross-sectional exploratory study aimed to detect major zoonotic agents in livestock during the GMT and to provide baseline evidence to guide One Health surveillance.

**Materials and methods:**

From 2022 to 2024, post-mortem samples (blood, nasal and rectal swabs, skin, ticks) were collected from sacrificed animals and analyzed using molecular and culture-based methods.

**Results:**

88 animals were included. *Borrelia* spp. was detected in 28.8 % of bovine blood samples, mostly in 2023 and 8.3 % of dromedaries. Enterohemorrhagic *Escherichia coli* occurred in 64.3 % of cattle and 33.3 % of dromedaries’ rectal samples. Nasal swabs showed *Mycobacterium* spp in 54.9 % bovines and 75.0 % dromedaries. Among hard ticks from bovines (predominantly *Hyalomma* spp.), 10.2 % and 11.5 %) were infected with *Borrelia* spp. and *Rickettsia* spp., including *R. aeschlimannii* and *R. sibirica mongolitimonae*. and one case of *Coxiella burnetii* was found. In dromedaries, only *Borrelia* spp. (43.7 %) was found in ticks. No dermatophytes were isolated.

**Conclusion:**

These findings demonstrate active zoonotic circulation and stress the need for continuous surveillance of zoonotic pathogens in animals involved in the GMT. Given the close proximity of humans and livestock during such events, the potential for spillover of zoonotic agents necessitates a *One Health* approach, integrating veterinary, environmental, and human health surveillance. Future investigations should further characterize pathogen species to better inform risk reduction strategies.

## 1 Introduction

Mass gatherings present significant public health challenges due to the potential for increased transmission of infectious diseases, particularly resulting from inter-human transmission due to overcrowding conditions [1,2]. Nevertheless, infections resulting from environmental pathogens are also a threat in this context [3]. Finally, because many animals are sacrificed in the specific context of certain religious mass gatherings, zoonoses may result from close human–animal interactions [4].

The Grand Magal of Touba (GMT), one of the largest annual religious pilgrimages in West Africa, attracts several million participants each year to the holy city of Touba, Senegal [5,6]. As part of the religious observance, large numbers of domestic animals, primarily cattle and dromedaries, are transported and sacrificed following Islamic rites and require veterinary inspection. As an example, about 5000 bovines, 7000 sheep, 1700 camels and 1500 goats were sold at the Touba market during the 2011 GMT [7]. This context offer unique opportunities for the emergence or re-emergence of zoonotic pathogens, in settings where animal veterinary screening is based on inspection only and where biosafety measures are limited [8,9]. During the 2025 GMT, over 2.5 million poultry, and 5053 bovines, 4178 sheep, 664 goats, and 130 camels were officially checked before consumption [10]. Veterinary oversight at the GMT starts at the borders of neighboring countries, such as Mauritania and Mali, where the Ministry of Agriculture, Food Sovereignty, and Livestock deploy officers to ensure the sanitary safety of animal-derived food products entering Senegal. Within Touba, mobile veterinary teams (approximately 41 agents in 2025) patrol the city and surrounding areas to prevent clandestine slaughter, monitor processed products, and ensure proper storage. These measures complement the household-level inspections performed before ritual sacrifice, collectively reducing public health risks.

While some studies have focused on zoonotic risks associated with the Hajj pilgrimage in Saudi Arabia [11], limited data are available on the circulation of zoonotic and enteric pathogens in animals sacrificed during the GMT [12]. This knowledge gap is particularly critical given the increased human exposure to blood, excreta, respiratory secretions, and ectoparasites during the sacrificial process. Pathogens such as *Borrelia* spp., *Brucella* spp., *Mycobacterium* spp., *Rickettsia* spp., and enteric bacteria (e.g., Enterohemorrhagic *Escherichia coli* (EHEC), *Salmonella*) are of particular concern due to their zoonotic potential and public health impact in sub-Saharan Africa [13,14].

The objective of this study was to assess the presence of zoonotic bacteria, viruses, parasites, and fungi in blood, nasal and rectal swab samples, ticks, and skin samples collected from cattle and dromedaries sacrificed during the GMT.

## 2 Materials and methods

### 2.1 Study design

Between 2022 and 2024, cattle and dromedaries sacrificed during the GMT were sampled at pilgrims’ homes in a cross-sectional study. Inclusion therefore depended on community acceptability, presence of the research team at the exact time of sacrifice, and the capacity to preserve and transport samples under appropriate conditions.

### 2.2 Veterinary control of animals

All animals included in our study underwent routine veterinary inspection prior to sacrifice. This included a clinical examination to verify that they were apparently healthy and fit for human consumption. Post-mortem inspection was also conducted on-site by municipal veterinary officers or qualified technicians.

### 2.3 Sampling

Cattle and dromedaries were sampled at pilgrims’ homes immediately after slaughtering. Inclusion required authorization from the household and confirmation that the carcass was intended for consumption. Specimen included blood in EDTA, nasal and rectal swabs, ticks manually removed from carcasses and skin scrapings from suspected lesions. Laboratory analyses of DNA/RNA were extracted with KingFisher Flex. qPCR assays were performed with controls (Ct ≤ 35 positive). Target panels included *Coxiella burnetii, Bartonella spp., Borrelia spp., Rickettsia spp., Anaplasma spp. Leptospira* spp. and *Brucella spp* for blood and tick samples*, Cryptosporidium* spp., *Mycobacterium* spp., *Salmonella* spp. hepatitis E virus, EHEC and *Giardia lamblia* for rectal samples*, Brucella* spp. and human coronaviruses for nasal samples. Skin samples were culture on Sabouraud agar medium.

### 2.4 Ethics statement

All samples were collected post-mortem from animals already designated for ritual sacrifice, and no animals were killed for research purposes. Sampling was conducted in accordance with Senegalese regulations, causing no additional distress to the animals. Municipal veterinary officers or qualified technicians were present during both pre- and post-mortem inspections to ensure animal welfare. The study protocol was reviewed and approved by the relevant institutional authorities, and all sampled animals were subsequently used for human consumption, consistent with local cultural and religious practices.

Full details of materials and methods are provided in the Supplementary Data.

## 3 Results

### 3.1 Context of sampling

A convenience sample of 88 animals was included with 15 bovines in 2022, 56 in 2023 and 5 in 2024 and 12 dromedaries in 2024. Animals included were proposed by households for ritual sacrifice and all appeared healthy at visual inspection. Sampling occurred in domestic settings outside formal abattoirs, with no systematic veterinary records; no gross lesions were observed. Animals belonged to individual families and were kept at the residence before slaughtering, allowing close human–animal contact. No information was available about the exact geographical origin of animals. The main livestock production area in Senegal is in the northeast of the country (Ferlo region) [15,16]. For major events such as the Tabaski and the GMT, live animals are often imported from neighboring countries, primarily Mauritania and Mali. Animals are then aggregated in Dahar and subsequently distributed to markets in Dakar, Saint Louis, Touba, Thiès, and Kaolack. While exact origins of animals in our study could not be determined, these movements illustrate the potential for livestock-mediated spread of zoonotic pathogens during mass gatherings. All sampled animals were subsequently consumed as part of the pilgrims’ meals.

### 3.2 Prevalence of zoonotic bacterial pathogens in blood samples collected from bovines and dromedaries

A total of 64 blood samples were analyzed, including 48 collected in 2023 and 16 in 2024, from 52 bovines and 12 dromedaries. Table 1 presents the detection of zoonotic bacterial pathogens in blood samples collected from bovines and dromedaries. Only *Borrelia* spp. was detected among the pathogens screened. In 2023, *Borrelia* spp. prevalence was 31.2 % (15 out of 48 bovines), while no detection was observed in 2024 among 4 animals, resulting in an overall prevalence of 28.8 % (15/52) across the study period. Specifically, in dromedaries sampled in 2024 (12 animals), *Borrelia* spp. was detected in one sample (8.3 %). No other zoonotic bacterial pathogens, including *Bartonella* spp., *C. burnetii*, *Anaplasma* spp., *Brucella* spp., *Rickettsia* spp., or *Leptospira* spp., were identified in any samples.

**Table 1:** Zoonotic bacterial pathogens in blood samples collected from bovines and dromedaries in 2023 and 2024.

	Bovines			Dromedaries
	2023 N = 48	2024 N = 4	Total N = 52	2024 N = 12
*Borrelia* spp.	15 (31.2 %)	0 (0 %)	15 (28.8 %)	1 (8.3 %)
*Bartonella* spp.	0 (0 %)	0 (0 %)	0 (0 %)	0 (0 %)
*Coxiella burnetii*	0 (0 %)	0 (0 %)	0 (0 %)	0 (0 %)
*Anaplasma* spp.	0 (0 %)	0 (0 %)	0 (0 %)	0 (0 %)
*Brucella* spp.	0 (0 %)	0 (0 %)	0 (0 %)	0 (0 %)
*Rickettsia* spp.	0 (0 %)	0 (0 %)	0 (0 %)	0 (0 %)
*Leptospira* spp.	0 (0 %)	0 (0 %)	0 (0 %)	0 (0 %)

### 3.3 Prevalence of zoonotic pathogens in rectal and nasal samples collected from bovines and dromedaries

Between 2022 and 2024, a total of 71 nasal and 70 rectal swab samples were collected from cattle, alongside 12 nasal and 12 rectal swab samples from dromedaries (Table 2).

**Table 2:** Zoonotic and enteric pathogens in rectal and nasal samples collected from bovines and dromedaries in 2022, 2023, and 2024.

	Bovines				Dromedaries
Rectal samples					
	2022 N = 10	2023 N = 55	2024 N = 5	Total N = 70	2024 N = 12

HEV	0 (0 %)	0 (0 %)	0 (0 %)	0 (0 %)	0 (0 %)
EHEC	7 (70 %)	36 (65.4 %)	2 (40 %)	45 (64.3 %)	4 (33.3 %)
*Mycobacterium* spp.	2 (20 %)	16 (29.1 %)	1 (20 %)	19 (27.1 %)	0 (0 %)
*Mycobacterium tuberculosis*	0 (0 %)	0 (0 %)	0 (0 %)	0 (0 %)	0 (0 %)
*Mycobacterium bovis*	0 (0 %)	0 (0 %)	0 (0 %)	0 (0 %)	0 (0 %)
*Salmonella* spp.	0 (0 %)	0 (0 %)	0 (0 %)	0 (0 %)	1 (8.3 %)
*Cryptosporidium* spp.	0 (0 %)	0 (0 %)	0 (0 %)	0 (0 %)	0 (0 %)
*Giardia lamblia*	1 (10 %)	0 (0 %)	0 (0 %)	1 (1.4 %)	0 (0 %)

Nasal samples					
	2022 N = 11	2023 N = 55	2024 N = 5	Total N = 71	2024 N = 12

HCoV	0 (0 %)	0 (0 %)	0 (0 %)	0 (0 %)	0 (0 %)
*Brucella* spp.	0 (0 %)	0 (0 %)	0 (0 %)	0 (0 %)	0 (0 %)
*Mycobacterium* spp.	3 (27.3 %)	35 (63.6 %)	1 (20 %)	39 (54.9 %)	9 (75 %)
*Mycobacterium tuberculosis*	0 (0 %)	0 (0 %)	0 (0 %)	0 (0 %)	0 (0 %)
*Mycobacterium bovis*	0 (0 %)	0 (0 %)	0 (0 %)	0 (0 %)	0 (0 %)

HEV: Hepatitis E Virus, EHEC: Enterohemorrhagic *Escherichia coli,* HCoV: Human Coronaviruses.

In bovine rectal samples (N = 70), EHEC was the most frequently detected pathogen, with a prevalence of 64.3 % (45/70). *Mycobacterium* spp. was found in 27.1 % (19/70) of these samples. Other pathogens such as *G. lamblia* were rare (1.4 %, 1/70), and no cases of HEV, *Salmonella* spp., *Cryptosporidium* spp., *M. tuberculosis*, or *M. bovis* were detected. In bovine nasal samples (N = 71), *Mycobacterium* spp. was detected in over half of the cases (54.9 %, 39/71), while *Brucella* spp., *M. tuberculosis*, and other tested pathogens were absent.

For dromedaries (N = 12), rectal samples showed the presence of EHEC in 33.3 % (4/12), and *Salmonella* spp. in 8.3 % (1/12) of samples. No other enteric pathogens were detected. Nasal samples from dromedaries revealed a high prevalence of *Mycobacterium* spp., found in 75.0 % (9/12) of samples, with no detection of *Brucella* spp., human coronaviruses, *M. tuberculosis*, or *M. bovis*.

### 3.4 Distribution of tick species collected from bovines and dromedaries

Ticks were opportunistically collected from 32 bovines between 2022 and 2024 (13 in 2022, 18 in 2023, and 1 in 2024) and from 11 dromedaries in 2024.

A total of 226 hard ticks were collected from bovines over the three-year period, of which 222 were successfully morphologically identified, while four specimens could not be identified due to damage or poor preservation (Table 3). The most prevalent species found on bovines were *Hyalomma truncatum* (55.4 %, 123/222), followed by *Rhipicephalus evertsi* (15.8 %, 35/222), *R. (Boophilus) microplus* (8.6 %, 19/222), *H. rufipes* (7.2 %, 16/222), *Amblyomma variegatum* (7.2 %, 16/222), *H. impeltatum* (4.1 %, 9/222), and *R. sanguineus* (0.9 %, 2/222). In dromedaries sampled in 2024, 48 hard ticks were identified (Table 3). The dominant species were *H. truncatum* (49 %) and *H. rufipes* (47.1 %), with a smaller proportion of *R. evertsi* (3.9 %). No other hard tick species were detected on dromedaries.

**Table 3:** Morphological identification of hard ticks species collected from bovines and dromedaries in 2022, 2023, and 2024.

	Bovines				Dromedarie
	2022 N = 140	2023 N = 83	2024 N = 3	Total N = 222	2024 N = 48
*Hyalomma truncatum*	59	64	1	123	24
*Hyalomma rufipes*	8	9	0	17	24
*Rhipicephalus evertsi*	31	2	2	35	0
*Amblyomma variegatum*	8	8	0	16	0
*Rhipicephalus (Boophilus) microplus*	19	0	0	19	0
*Hyalomma impeltatum*	9	0	0	9	0
*Rhipicephalus sanguineus*	2	0	0	2	0
Not identified	4	0	0	0	0

### 3.5 Prevalence of zoonotic bacterial pathogens in ticks

A total of 226 hard ticks collected from cattle between 2022 and 2024 and 48 hard ticks from dromedaries in 2024 were tested by qPCR for zoonotic bacterial pathogens (Table 4).

**Table 4:** Zoonotic bacterial pathogens in hard ticks collected from bovines in 2022, 2023, and 2024.

	Hard ticks from bovines				Hard ticks from dromedaries
	2022 N = 140	2023 N = 83	2024 N = 3	Total N = 226	2024 N = 48
*Borrelia* spp.	12 (8.6 %)	9 (10.8 %)	2 (66.7 %)	23 (10.2)	21 (43.7)
*Bartonella* spp.	0 (0 %)	0 (0 %)	0 (0 %)	0 (0 %)	0 (0 %)
*Coxiella burnetii*	1 (0.7 %)	0 (0 %)	0 (0 %)	1 (0.4 %)	0 (0 %)
*Anaplasma* spp.	0 (0 %)	0 (0 %)	0 (0 %)	0 (0 %)	0 (0 %)
*Brucella* spp.	0 (0 %)	0 (0 %)	0 (0 %)	0 (0 %)	0 (0 %)
*Rickettsia* spp.	19 (13.5 %)	7 (10 %)	0 (0 %)	26 (11.5 %)	0 (0 %)
*Rickettsia conorii conorii*	0 (0 %)	0 (0 %)	0 (0 %)	0 (0 %)	0 (0 %)
*Rickettsia aeschlimannii*	8 (5 %)	5 (6.0 %)	0 (0 %)	13 (5.7 %)	0 (0 %)
*Rickettsia sibirica mongolitimonae*	3 (1.8 %)	0 (0 %)	0 (0 %)	3 (1.3 %)	0 (0 %)
*Rickettsia massiliae*	0 (0 %)	0 (0 %)	0 (0 %)	0 (0 %)	0 (0 %)
*Leptospira* spp.	0 (0 %)	0 (0 %)	0 (0 %)	0 (0 %)	0 (0 %)

Among the hard ticks from cattle, *Borrelia* spp. and *Rickettsia* spp. were the most frequently detected bacteria, with an overall prevalence of 10.2 % (23/226) and 11.5 % (26/226), respectively. *Borrelia* spp. were found in *H. truncatum, H. rufipes* and *A. variegatum.* The detection rate of *Borrelia* spp. varied by year: 8.6 % (12/140) in 2022, 10.8 % (9/83) in 2023, and 66.7 % (2/3) in 2024. *Rickettsia* spp. was found in 13.5 % (19/140) of ticks in 2022 and 8.4 % (7/83) in 2023, in *H. rufipes*, *A. variegatum* and *H. truncatum*. Species-level identification revealed the presence of *R. aeschlimannii* in 5.7 % tick (13/226) in *H. rufipes, A. variegatum* and *H. truncatum* and *R. sibirica mongolitimonae* in 1.3 % ticks (3/226) in *A. variegatum*. One case of *C. burnetii* was observed (1/226, 0.4 %) in *H. truncatum*. No PCR was positive for *Bartonella* spp., *Anaplasma* spp., *Brucella* spp., *Leptospira* spp., or other *Rickettsia* species.

In ticks collected from dromedaries, only *Borrelia* spp. was detected, with a high prevalence of 43.7 % (21/48) in *H. truncatum* and *H.rufipes*, while all other tested pathogens were absent.

### 3.6 Proportion of animals with infected ticks

Out of 274 hard ticks collected from 32 tick-infested cattle and 11 tick-infested dromedaries, *Borrelia* spp. was the most frequently detected pathogen, with 44 positive ticks. These infected ticks were found on 14/32 cattle (43.8 %) and 9/11 dromedaries (81.8 %), corresponding to the proportion of tick-infested animals carrying at least one *Borrelia*-positive tick. *C. burnetii* was detected in one tick on 1/32 bovine (3.1 %). *Rickettsia* spp. was detected in 26 ticks, all collected from cattle, involving 12/32 tick-infested cattle (37.5 %). Further species-level identification revealed *Rickettsia aeschlimannii* in ticks collected from 7/32 cattle (21.9 %) and *Rickettsia sibirica mongolitimonae* in ticks from 1/32 cattle (3.1 %). No Rickettsia species were detected in hard ticks collected from dromedaries (Table 5).

**Table 5:** Total number of bovines and dromedaries carrying infected hard ticks by pathogen (2022–2024).

Detected pathogen	Number of bovines carrying infected ticks N = 32	Number of dromedaries carrying infected ticks N = 11
*Borrelia* spp.	14 (43.7 %)	9 (81.8)
*Coxiella burnetii*	1 (3.1 %)	0 (0 %)
*Ricketssia* spp.	12 (37.5)	0 (0 %)
*Rickettsia aeschlimannii*	7 (21.9)	0 (0 %)
*Rickettsia sibirica mongolitimonae*	1 (3.1)	0 (0 %)

### 3.7 Detection of dermatophytes in skin samples

71 animals presented with skin lesions possibly suggestive of dermatophytosis. No dermatophyte species were detected in any of the skin samples collected from cattle in 2022 and 2023.

## 4 Discussion

Religious mass gatherings such as the GMT represent unique ecological and epidemiological settings where the interface between humans, animals, and vectors may facilitate the emergence and transmission of zoonotic pathogens. Understanding the circulation of bacterial zoonoses within livestock involved in such events is critical to assess public health risks and to design effective control strategies. In this context, our study provides important insights into the prevalence and diversity of zoonotic bacterial pathogens circulating among cattle and dromedaries sacrificed during the GMT between 2022 and 2024.

Our study provides compelling evidence of *Borrelia* spp. prevalence in both bovines and dromedaries in Senegal, suggesting that these animals serve as significant hosts for *Borrelia*-infected ticks. The detection of *Borrelia* spp. in 28.8 % of bovine blood samples and 8.3 % of dromedary blood samples, coupled with a high prevalence of 43.7 % (21/48) in hard ticks collected from dromedaries and 10.2 % (23/226) in bovines, underscores the active circulation of these pathogens. Given that the ticks in our study are hard ticks, it is unlikely that the detected *Borrelia* species are *B. crocidurae*, which is typically transmitted by soft ticks (*Ornithodoros* spp.) [17]. Instead, *B. theileri*, a species associated with hard ticks, is a more probable candidate. *B. theileri* has been reported as the causative agent of bovine borreliosis in livestock, including cows, goats, and sheep, and is transmitted by various hard tick species, primarily within the genus *Rhipicephalus* [18,19]. Furthermore, *B. theileri* has been detected in tick blood collected from cattle in regions such as Argentina and Cameroon, indicating its presence in diverse geographical areas. This supports the notion that hard ticks infesting bovines and dromedaries in Senegal could be vectors for *B. theileri* [20,21]. A study conducted in southwestern Ethiopia reported the detection of a *Borrelia* sp. in *A. cohaerens* that was genetically distinct from known pathogenic species such as *B. duttonii* and *Borrelia* detected in *Ornithodoros porcinus*, showing only 84–86 % identity. Phylogenetic analysis suggested that this bacterium occupies an intermediate position between the recurrent fever and Lyme disease groups, potentially representing a third, previously unrecognized clade of the genus. These findings underscore the genetic diversity of *Borrelia* circulating in hard ticks in Africa and highlight the need for further investigation into their potential role in human and animal disease [22].

Regarding *Rickettsia* spp., these obligatory intracellular bacteria are responsible for numerous tick-borne diseases worldwide. The detection of *R. aeschlimannii* and *R. sibirica mongolitimonae* in ticks from cattle in our study is consistent with their documented geographic distribution in Africa and their established association with *Hyalomma* ticks. *R. aeschlimannii* has emerged as a human pathogen in Mediterranean and African regions and is transmitted mainly by *Hyalomma* spp., causing spotted fever group rickettsioses. The absence of *Rickettsia* spp. in ticks collected from dromedaries may be due to host-vector-pathogen specificities or ecological factors, warranting further research into these interactions [23].

The detection of a single tick positive for *C. burnetii* among hard ticks collected from cattle in Senegal suggests that the pathogen is present at a low prevalence in this environment. A study identified *C. burnetii* in hard and soft ticks collected from domestic animals in rural Senegal, with infection rates ranging from 0 % to 37.6 %, indicating that while *C. burnetii* is present in tick populations in Senegal, the prevalence can vary [24].

The absence of other major zoonotic bacteria such as *Bartonella* spp., *Brucella* spp., and *Leptospira* spp. may reflect either low prevalence in these populations or limitations of detection in the sample size and methods used.

Similarly, enteric pathogen analysis highlighted a high prevalence of EHEC in bovine rectal swabs (64.3 %), consistent with cattle as recognized reservoirs of EHEC strains causing human illness worldwide [25]. These findings are further supported by epidemiological data from the GMT, where prospective cohort surveys showed that 17.8 % of pilgrims acquired EHEC during or shortly after the pilgrimage. This underscores the zoonotic potential of EHEC strains and highlights the public health risks associated with livestock-human-environment interactions during mass gatherings [26].

In this study, *Mycobacterium* spp. was detected in a substantial proportion of bovine and dromedary samples, with prevalence rates of 27.1 % in bovine rectal samples, 54.9 % in bovine nasal samples, and 75 % in dromedary nasal samples. Notably, neither *M. bovis* nor *M. tuberculosis* was detected in any of the samples. For instance, studies conducted in Africa indicate that zoonotic tuberculosis accounts for a small proportion of human tuberculosis cases, generally ranging from <1 % in some regions to 5 % in others [27]. In Senegal specifically, data on *M. bovis* infections in humans or animals are limited, but reports suggest sporadic detection and low endemicity compared to countries like Tanzania, where up to 16 % of human tuberculosis cases are attributed to *M. bovis* [28]. Although *M. bovis* was not detected in our bovine and dromedary samples, the presence of *Mycobacterium* spp., particularly in nasal swabs, highlights a potential reservoir in the upper respiratory tract. In Senegal, as in many developing countries, bovine tuberculosis remains poorly documented in both humans and animals, and conventional control methods are limited. The absence of *M. bovis* in our study aligns with the low reported prevalence in livestock in West Africa, but surveillance is essential given the endemic nature of bovine tuberculosis in some regions and the risk to humans, especially those immune-compromised. The development of vaccines for livestock and improved diagnostic tools, as discussed in global studies, would strengthen control efforts and help prevent zoonotic transmission [29].

Our study has some limitations. Sample sizes, particularly for dromedaries and ticks in 2024, were limited due to the logistical challenges of accessing household-level slaughter, coordinating personnel and equipment, and preserving samples. Thus, results reflect detection of frequencies in an opportunistic sample and cannot be generalized to all animals sacrificed at the GMT. Molecular assays did not always allow species-level identification for some pathogens; the cross-sectional design limits inference on transmission dynamics, not all zoonotic agents were assessed, and the geographic origin of animals was not documented.

Overall, these data underscore the importance of continuous surveillance of zoonotic pathogens in animals involved in human mass gatherings, both to better understand pathogen ecology and to inform targeted interventions reducing risks to human health. Given the proximity of humans and livestock during such events, the potential for spillover of zoonotic agents necessitates a *One Health* approach, integrating veterinary, environmental, and human health surveillance. Moreover, the handling of livestock carcasses outside formal abattoirs during the GMT often occurs under informal conditions, potentially increasing human exposure to blood and tissues contaminated with zoonotic pathogens, and further emphasizing the need for preventive measures and hygiene awareness. In addition, our findings highlight the need to raise awareness among households and pilgrims about tick-bite prevention, given the frequent detection of zoonotic pathogens in ticks. Future investigations should also focus on human-specific pathogens and further characterize pathogen species to better inform risk reduction strategies.

## 5 Conclusion

Our study highlights the active circulation of several zoonotic bacterial pathogens among livestock and their vectors during the GMT, with particular emphasis on *Borrelia* spp. and tick-borne pathogens. These findings provide essential baseline data to guide surveillance and control efforts in such high-risk settings. Despite its limitations, this work contributes valuable insights into the epidemiology of zoonotic bacterial pathogens in livestock at mass gatherings, reinforcing the need for integrated One Health surveillance programs to mitigate zoonotic risks. Future investigations should focus on additional human-specific pathogens.

## CRediT authorship contribution statement

**Ihssane Ouaddane:** Writing – original draft, Investigation, Formal analysis. **Coumba Diouf:** Investigation, Formal analysis. **Georges Diatta:** Methodology, Investigation. **Adama Zan Diarra:** Formal analysis. **Mbayang Faye:** Investigation. **Cheikh Sokhna:** Writing – review & editing, Conceptualization. **Philippe Gautret:** Writing – review & editing, Validation, Supervision, Methodology, Conceptualization.

## Ethics

All animal samples were collected post-mortem following ritual slaughter and required veterinary inspection, during the GMT. The protocol was approved by the Senegalese National Ethics Committee for Health Research (SEN17/62).

Authorization for international sample transfer was granted by the Senegalese authorities under permit number 000410 DPN/PF-APA and approved by French authorities (Ref.ER27-2025).

## Funding

This research was funded by the Institut Hospitalo-Universitaire (IHU) Méditerranée Infection, the 10.13039/501100001665French National Research Agency under the “Investissements d'avenir” programme, reference ANR-10-IAHU-03**.**

## Declaration of competing interest

The authors declare that they have no known competing financial interests or personal relationships that could have appeared to influence the work reported in this paper.
