# Supplementary material for: Surveillance of zoonotic pathogens in cattle and dromedaries sacrificed at the Grand Magal of Touba: a cross-sectional survey
Source: New Microbes New Infect. 2026 Jan 27;70:101714. doi: 10.1016/j.nmni.2026.101714 (PMC12874613; doi:10.1016/j.nmni.2026.101714)
Supplement: Multimedia component 1 [file mmc1.docx]

**Supplementary Data**

# **Materials and Methods**

## ***Study site and target population***

The study was conducted during the GMT from 2022 to 2024. The target population included cattle and dromedaries brought for sacrifice. Animals were sampled at the homes of pilgrims who performed the sacrifices, following traditional practices and required veterinary inspection. This approach ensured the inclusion of animals directly linked to the religious activities of the event.

## ***Blood sampling***

Blood samples were collected from the neck vessels of animals directly after the sacrifice, in accordance with Islamic sacrificial practices. The blood was slowly shaken and immediately stored into sterile EDTA tubes to ensure sample integrity.

## ***Nasal and rectal swab collection***

Nasal and rectal swabs samples were collected from each animal post-mortem to detect potential pathogens. Samples were taken using commercial rigid cotton swab applicators (Medical Wire & Equipment, Wiltshire, UK, MW176S), inserted into the nostrils of animals, and the swabs were rotated gently for 5-10 seconds to ensure adequate sample collection. Rectal swabs were obtained with sterile swabs inserted into the rectum of each animal and rotated gently to collect fecal material. The sample was then placed in a viral transport medium (Sigma Virocult).

## ***Tick collection***

Ticks were manually removed post-mortem, from the animals' skin. The ticks were placed in a sterile nunc containing 90% ethanol for preservation. Ticks were identified in the laboratory using a stereomicroscope and taxonomic keys [1].

## ***Skin sampling***

Skin samples were collected from dead animals to assess the presence of dermatophyte infections. For each animal, a sterile gauze pad moistened with sterile saline was gently rubbed over areas of the skin showing clinical signs suggestive of dermatophytosis (such as alopecia, crusts, or scaling). The gauze was then placed into a sterile tube.

## ***Sample storage and transportation***

Swab samples were maintained at 4°C immediately after collection and transported to the laboratory in Dakar, where they were stored in a −80 °C freezer. They were then transferred to Marseille on dry ice for further processing. Ticks stored in sterile nunc tubes containing 90°C ethanol and skin samples were kept at room temperature until processing.

## ***Identification of pathogens***

DNA and RNA were extracted with the KingFisher™ Flex system. Quantitative real-time PCR assays were conducted using a C1000 Touch™ Thermal Cycler (Bio-Rad, Hercules, CA, USA). Amplifications were performed with the LightCycler® 480 Probes Master kit (Roche Diagnostics, France), in accordance with the manufacturer's instructions. Each run included both negative controls (PCR mix) and positive controls (DNA from bacterial or parasitic strains or RNA from viral strains). A cycle threshold (CT) value of ≤ 35 was considered indicative of a positive result for bacterial, parasitic, or viral amplification. Primers used are described in Supplementary Table 1.

The selection of pathogens tested was based on the most well-known zoonoses that are transmitted from animals to humans, with the choice tailored to the type of samples collected [2].

In blood samples, *Coxiella burnetii*, *Bartonella* spp., *Borrelia* spp.*, Rickettsia* spp., *Anaplasma* spp., and *Brucella* spp. were investigated.

For rectal samples, pathogens including HEV (Hepatitis E Virus), Enterohemorrhagic *Escherichia coli* (EHEC), *Leptospira* spp., *Cryptosporidium* spp., *Mycobacterium* spp., *M. tuberculosis, M. bovis, Salmonella* spp., and the 18S gene *Giardia lamblia* were identified.

Nasal samples were tested for the presence of Human Coronaviruses (HCoV*)* using one-step duplex quantitative RT-PCR amplifications with the HCoV/HPIV-R Gene Kit (REF: 71-045, BioMérieux, Marcy l’Etoile, France), following the manufacturer's recommendations and for *Brucella* spp.

Tick samples were tested for *C. burnetii*, *Bartonella* spp*.*, *Borrelia* spp*.*, *Rickettsia spp.*, *R. conorii conorii*, *R. aeschlimannii*, *R. sibirica mongolitimonae*, *R. massiliae*, and *Anaplasma* spp*.* Each tick tube was labeled with the animal’s identification number, the collection site, and the date of sampling.

Additionally, cutaneous samples from cattle collected using sterile gauze swabs were treated on Sabouraud dextrose agar supplemented with chloramphenicol and gentamicin, to investigate the presence of dermatophytes.

**Supplementary table 1.** Primers and probes for qPCR testing

| Pathogens | Target sequence | Primers/Probes | Sequence (5’ 🡪 3’) |
| --- | --- | --- | --- |
| *Bartonella* spp. | ITS2 | Bart-ITS2_F | GGGGCCGTAGCTCAGCTG |
|  |  | Bart-ITS2_R | TGAATATATCTTCTCTTCACAATTTC |
|  |  | Bart-ITS2_P | FAM-CGATCCCGTCCGGCTCCACCA-TAMRA |
| *Borrelia* spp. | 16S | Bor16S-F | AGCCTTTAAAGCTTCGCTTGTAG |
|  |  | Bor16S-R | GCCTCCCGTAGGAGTCTGG |
|  |  | Bor16S-P | FAM-CCGGCCTGAGAGGGTGAACGG-TAMRA |
| *Coxiella burnetii* | IS30A | CB_IS30A_3F | CGCTGACCTACAGAAATATGTCC |
|  |  | CB_IS30A_3R | GGGGTAAGTAAATAATACCTTCTGG |
|  |  | CB_IS30A_3P | 6FAM-CATGAAGCGATTTATCAATACCTGTATGC-TAMRA |
| *Anaplasma* spp. | 23S rRNA | Ttana-R | GTAACAGGTTCGGTCCTCCA |
|  |  | Ttana-F | TGACAGCGTACCTTTTCGAT |
|  |  | Ttana-P | 6FAM-GGATTAGACCCGAAACCAAG-TAMRA |
| *Rickettsia* spp. | citrate synthase (gltA) | RKNDO-F | GTGAATGAAAGATTACACTATTTAT |
|  |  | RKNDO-R | GTATCTTAGCAATCATTCTAATAG |
|  |  | RKNDO-P | FAM-CTATTATGCTTGCGGCTGTCGGTTC-TAMRA |
| *Rickettsia conorii conorii* | hypothetical protein  RC0743 | Rcono7_F | TTGGTAGGCAAGTAGCTAAGCAAA |
|  |  | Rcono7_R | GGAAGTATATGGGAATGCTTTGAA |
|  |  | Rcono7_P | 6FAM- GCGGTTATTCCTGAAAATAAGCCGGCA |
| *Rickettsia aeschlimannii* | sca1 | Raes_sca1_F | AAGCGGCACTTTAGGTAAAGAAA |
|  |  | Raes_sca1_R | CATGCTCTGCAAATGAACCA |
|  |  | Raes_sca1_P | 6FAM- TGGGGAAATATGCCGTATACGCAAGC |
|  | sca2 | Raes_sca2_F | AGCCTGCCAAATTCTCTCAA |
|  |  | Raes_sca2_R | TGGAAATTATTGAAGATTCTCCTTT |
|  |  | Raes_sca2_P | 6FAM- GCAAAGACGATTGGTATACGTGGTCAAA |
| *Rickettsia sibirica mongolitimonae* | Unknow | Rmon_F4 | AGGGTAAAGTACTTGCTGAATCA |
|  |  | Rmon_R4 | TGTAGCGCCGGTAAATGATAA |
|  |  | Rmon_P4 | 6FAM- GAGCCTCGCGAAATTTGCAA -MGB |
| *Rickettsia massiliae* | hypothetical protein | Rmas_9666_F | CCAACCTTTTGTTGTTGCAC |
|  |  | Rmas_9666_R | TTGGATCAGTGTGACGGACT |
|  |  | Rmas_9666_P | 6FAM- CACGTGCTGCTTATACCAGCAAACA |
| *Leptospira* spp. | Lip32 | Lip32L32-Rb | GAACTCCCATTTCAGCGAT |
|  |  | LipL32-45F | AAGCATTACCGCTTGTGGTG |
|  |  | Lip32_189P | FAM-AAAGCCAGGACAAGCGCCG-TAMRA |
| HEV | capsid protein gene | HEV_F | GGTGGTTTCTGGGGTGAC |
|  |  | HEV_R | AGGGGTTGGTTGGATGAA |
|  |  | HEV_P | 6FAM- TGATTCTCAGCCCTTCGC |
| EHEC | Stx1 | slt1-1 for | CTT CCA TCT GCC GGA CAC ATA |
|  |  | slt1-2 rev | ATT AAT ACT GAA TTG TCA TCA TCA TGC AT |
|  |  | Probe | VIC-AAG GAA ACT CAT CAG ATG CCA TTC TGG CA- TAMRA |
|  | Stx2 | slt2-1 for | GAC GTG GAC CTC ACT CTG AAC TG |
|  |  | slt2-2 rev | TCC CCA CTC TGA CAC CAT CC |
|  |  | Probe | 6FAM -TAC TCC GGA AGC ACA TTGCTG ATT CGC-TAMRA |
| *Mycobacterium* spp. | ITS | IST-mycob F | GGGTGGGGTGTGGTGTTTGA |
|  |  | IST-mycob R | CAAGGCATCCACCATGCGC |
|  |  | IST-mycob P | 6FAM-TGGATAGTGGTTGCGAGCATC-TAMRA |
| *Mycobacterium tuberculosis* | ITS | Mtub_ITS_F | GGTGGGGTGTGGTGTTTGAG |
|  |  | Mtub_ITS_R | CAAGGCATCCACCATGCGC |
|  |  | Mtub_ITS_P | 6FAM- GCTAGCCGGCAGCGTATCCAT |
| *Mycobacterium bovis* | RD4 | R | AAGATCGACCCGCAGTG |
|  |  | F | GCAGAAGCGCAACACTCTTG |
|  |  | P | ATCTTAGCTGGTCAATAGCCATTTTT |
| *Salmonella* spp. | invA | invA_F | CTC-ACC-AGG-AGA-TTA-CAA-CAT-GG |
|  |  | invA_R | AGC-TCA-GAC-CAA-AAG-TGA-CCA-T C |
|  |  | Probe | HEX-CAC-CGA-CGG-CGA-GAC-CGA-C TT-T-BHQ1 |
| *Cryptosporidium* spp. | hsp70 gene | 1PS_F | AACTTTAGCTCCAGTTGAGAAAGTACTC |
|  |  | 1PS_R | CATGGCTCTTTACCGTTAAAGAATTCC |
|  |  | Probe | 6FAM – AATACGTGTAGAACCACCAACCAATACAACATC- TAMRA |
| *Giardia lamblia* | 18S | Giardia-80F | TTGCCAGCGGTGTCCG |
|  |  | Giardia-127R | GACGGCTCAGGACAACGGTT |
|  |  | Probe | FAM-CCCGCGGCGGTCCCTGCTAG-TAMRA |

**References cited only in Supplementary Data**

[1] Walker AR, Bouattour A, Camicas JL, Estrada-Peña A, Horak IG, Latif AA, Pegram RG, Preston PM, 2003. Ticks of domestic animals in Africa: a guide to identification of species. Press. Bioscience Report, Edinburgh

[2] Eldin, C., Mélenotte, C., Mediannikov, O., Ghigo, E., Million, M., Edouard, S., Drancourt, M., & Raoult, D. (2017). *From Q fever to Coxiella burnetii infection: a paradigm change*. Clinical Microbiology Reviews, 30(1), 115-190.
